# Potential Activity of Albino *Grifola frondosa* Mushroom Extract against Biofilm of Meticillin-Resistant *Staphylococcus aureus*

**DOI:** 10.3390/jof7070551

**Published:** 2021-07-10

**Authors:** Teresa Fasciana, Maria Letizia Gargano, Nicola Serra, Elena Galia, Ignazio Arrigo, Maria Rita Tricoli, Orazia Diquattro, Giuseppa Graceffa, Salvatore Vieni, Giuseppe Venturella, Anna Giammanco

**Affiliations:** 1 Department of Health Promotion, Mother and Child Care, Internal Medicine and Medical Specialties, University of Palermo, 90127 Palermo, Italy; anna.giammanco@unipa.it; 2 Department of Agricultural and Environmental Science, University of Bari Aldo Moro, Via Amendola 165/A, 70126 Bari, Italy; marialetizia.gargano@uniba.it; 3 Italian Society of Medicinal Mushrooms, 50121 Pisa, Italy; giuseppe.venturella@unipa.it; 4 Department of Public Health, University Federico II of Naples, 80138 Napoli, Italy; nicola.serra5@gmail.com; 5 A.O.U.P., Unit of Microbiology, Virology and Parasitology, 90127 Palermo, Italy; elenagalia9@gmail.com (E.G.); igni90@hotmail.it (I.A.); mariaritatricoli@gmail.com (M.R.T.); 6 Laboratory of Microbiology, Azienda Ospedaliera Ospedali Riuniti “Villa Sofia-V. Cervello”, 90127 Palermo, Italy; orazia.diquattro@villasofia.it; 7 Department of Surgical Oncological and Oral Sciences, University of Palermo, 90133 Palermo, Italy; giuseppa.graceffa@unipa.it (G.G.); salvatore.vieni@unipa.it (S.V.); 8 Department of Agricultural, Food and Forest Sciences, University of Palermo, 90133 Palermo, Italy

**Keywords:** *Grifola frondosa*, biofilm, *Staphylococcus aureus*, activities

## Abstract

Mushroom extracts are a rich source of natural compounds with antimicrobial properties, which are able to prevent, to some extent, the growth of foodborne pathogens. The aim of this study was to investigate the potential of extracts from albino *Grifola frondosa* (GF), commonly known as maitake, to inhibit the growth of some bacteria and the biofilm production by *Staphylococcus aureus.* We obtained not only a significant reduction of OD score between biofilm and biofilm plus albino *G. frondosa* extract group, but also a reduction of category of biofilm. In addition, we observed a significant presence of isolates with strong category for the biofilm group and a significant presence of isolates with absent category for the biofilm plus albino *G. frondosa* extract group. These results confirm that the use of albino *G. frondosa* extract reduces in significant way the presence of biofilm. Our results suggest and confirm that albino *G. frondosa* extracts could be employed as functional food and could be used as a natural additive for food process control and food safety.

## 1. Introduction

Natural compounds present in herbs and spice-derived extracts, essential oils and other secondary metabolites from plants, bacteria and enzymes are currently gaining ground and are still largely underused.

In recent years, there has been an increased interest in natural antimicrobials, especially those obtained from medicinal mushrooms [1]. Some fungi species are a rich source of natural compounds with antimicrobial properties, which can inhibit the growth of foodborne pathogens, and increase the sill life of the food [2].

Microorganisms present in foodborne diseases or food processing plant contamination are often capable of adhering and forming biofilm [3]. Biofilm is defined as a structured community of microbial cells firmly attached to a surface and embedded in a matrix composed of extracellular polymeric substances (EPS) [4]. The development of sessile biofilm communities involves highly complex and dynamic events whereby EPS play key structural and functional roles that are essential for the emergent properties of biofilms. EPS promote microbial adhesion to biotic and abiotic surfaces [5]. It is recognized that bacteria within biofilms are more resistant to antibiotics and other chemical agents than planktonic cells in suspension, and their increased tolerance towards antimicrobial agents reduces the effectiveness of treatment of biofilm-related infections [6]. Bacterial biofilms spread widely and play important roles in many industrial activities [7]. In the dairy industry or other food processing industries or food-contact surfaces, biofilm formation is a potential source of contamination and can lead to serious hygiene problems and economic losses [8,9]. 

Searching for new strategies to control this complex mode of bacterial life is extremely challenging [10]. Edible mushrooms are widely acknowledged for their nutritional and medicinal properties; however, their potential market is still far from being fully developed considering that there is a high consumer demand [11].

There have been several studies reporting the beneficial effects of *G. frondosa* in the treatment of viral infections, including those caused by hepatitis B virus (HBV), enterovirus 71 (EV71), herpes simplex virus type 1 (HSV-1) and human immunodeficiency virus (HIV). Patients who took *G. frondosa* fruiting body polysaccharides showed positive signs, specifically a higher recovery rate compared with the control group [12]. In addition to antiviral effects, the components isolated from *G. frondosa* have also shown antibacterial effects [12,13]. The extracts could activate immuno-competent cells and induce the production of cytokines, which further lead to the activity enhancement of splenic T cells in killing *Listeria monocytogenes* [13].

The new species of editable mushrooms with antibacterial activity, such as the albino *G. frondosa*, have shown promise [14].

The albino maitake mushroom is quite rare and distributed in temperate regions of North America, Europe, and Asia (Japan and China). Recently, it was also collected for the first time in Italy (Sicily, Southern Italy) [15,16]. A field investigation carried out in Sicily led to the collection of an unusual white *Grifola* specimen at the base of a living tree of Quercus pubescens Willd. s.l. [14,15].

Albino *G. frondosa* (Dicks.) Gray “maitake” mushrooms (described as *G. albicans* Imazeki and then placed in synonymy with *G. frondosa*) are particularly rare, and the few pertinent records are not treated in scientific publications [14].

The sequence of ITS (internal transcribed spacer) obtained by Gargano et al. is the only one available from an albino *Grifola* specimen from Italy; the rest of the *G. frondosa* sequences derive almost exclusively from east Asia (China, Japan, South Korea, Taiwan) and the USA [15].

However, most of the antibacterial and biofilm activities of medicinal mushrooms have been found in *Pleurotus* species, exhibiting activity against Gram-positive and Gram-negative bacteria [17,18]. 

The purpose of this paper is show for the first time that the extract of albino *G. frondosa*, isolated from tree of Quercus pubescens Willd. s.l., exhibits antibiofilm activity against methicillin-resistant *S. aureus* (MRSA) obtained from clinical samples. The current knowledge on its potential antibiofilm activities was reported only against ATCC strains [14].

## 2. Materials and Methods 

### 2.1. Characterization Albino G. frondosa 

The albino *G. frondosa* strain was harvested in Castelbuono forest on 10 January 2016. The collected basidioma was dried and then deposited in the Herbarium of the Department of Agricultural, Food, and Forest Sciences (SAAF 450). Morphological, molecular/phylogenetic, ecological data, information on the nutritional value and antimicrobial activity about this rare albino *Grifola* specimen were reported in our previously study described by Gargano et al. [14]. 

### 2.2. Bacterial Isolates and Growth Conditions 

The clinical strains of MRSA (*n* = 49) were recovered from the clinical samples, reported in Table 1, of hospitalized patients at the “Azienda Ospedaliera Ospedali Riuniti “Villa Sofia-V. Cervello”. The profile of antibiotic resistance of the strains is presented in supplementary file (File S1). The strains were identified by BD Phoenix™ (Becton Dickinson Europe Holdings SAS-Francia, Pont-de-Claix, France) and 16 s rRNA gene sequencing. Isolates were cultured on Columbia agar with 5% sheep blood for routine maintenance and storage. The bacterial strain ATCC 43300 was used as control [19].

### 2.3. Quantification of Biofilm Formation

Crystal violet assay (CVA) was executed to quantify biofilm formation by all 49 clinical isolates of MRSA and was further categorized into strong, moderate, or weak biofilm producers according to Calà et al. 2015 [16]. 

In brief, 100 µL of overnight grown culture (0.5 McFarland in tryptosebroth (BT)) was added to 100 µL of sterile BT in a sterile 96-well flat bottom microtiter plate (Biosigma S.r.l. a Dominique Dutscher Company, Venice, Italy) and incubated at 37 °C for 24 h. The assay was performed in triplicates. BT without bacterial culture was used as negative control for biofilm formation and ATCC 43300 of *S. aureus* strain was taken as a positive control [20]. 

After 24 h of incubation, the excess stain was washed away by flushing the wells with 0.85% saline twice and the adhered biofilm was fixed by adding 250 µL of methanol for 15 min. The biofilm formed was then stained by 250 µL of 0.5% crystal violet solution for 15 min. The stain attached to adherent layers was re-solubilized in 250 µL of 33% acetic acid for 15 min. The optical density (OD) of the solution was measured at 570 nm using a microtiter plate reader (Multiskan Go, Thermo Fisher Scientific, Waltham, MA, USA). The isolates were categorized into weak, moderate, or strong biofilm producers based on the cut-off OD. Cut-off OD (ODc) is defined as three standard deviations above mean OD of negative control (at 570 nm). Isolates were classified as follows: OD ≤ ODc = no biofilm producer, O.Dc < O.D. ≤ 2 × ODc) = weak biofilm producer, 2 ODc < OD ≤ (4 × ODc) = moderate biofilm producer, (4 × ODc) < OD = strong biofilm producer [20]. 

### 2.4. Biofilms Susceptibility Testing

Clinical strains and *S. aureus* ATCC 43300 were grown, diluted and wells were washed as previously described for the (CVA) method. The plates were air-dried at 37 °C and to each well was added 100 µL of BT supplemented with 25% extract, except in the case of positive controls. The plates were incubated at 37 °C for 24 h; after this incubation time, the biofilm with medium was removed, the plates were air-dried and then each well was filled with crystal violet solution (0.5%) for 15 min. The plate was then washed three times with water, and the crystal violet was dissolved in 250 µL of ethanol by pipetting up and down. The plate was read at 570 nm using a microplate reader. Inhibition percentages at concentrations of extract were obtained by comparing the OD of control wells with that of the sample wells (Di Domenico et al., 2016) [21]. 

### 2.5. Statistical Analysis

Statistical analysis was performed using the Matrix Laboratory (MATLAB) analytical toolbox version 2008 (MathWorks, Natick, MA, USA). Data are presented as number and percentage for categorical variables, and numerical data were expressed as the mean ± standard deviation (SD) or median with confidence interval (CI) at 95%. In addition, the OD values and the category for all isolates for the biofilm and biofilm plus albino *G. frondosa* extract (biofilm + GF) groups we defined according to the following classification: Absent (OD ≤ 0.078): category 1, Weak (0.078 < OD ≤ 0.156): category 2, Moderate (0.156 < OD ≤ 0.31): category 3, and Strong (OD > 0.31): category 4. In this way we considered for Biofilm and biofilm+ GF group the Global category variable, composed of all categories associated with all isolates considered. The test for normality was performed with the chi-square test. In this case, with a *p*-value < 0.05, the hypothesis of data normality was rejected. The Wilcoxon test was performed to evaluate significant difference between two paired samples. Finally, all *p*-values were always two-sided and all tests with *p*-value (*p*) < 0.05 were considered significant.

## 3. Results

In Table 2 we show all percentages of biofilm and biofilm+ GF group. In particular, we describe the percentages for every category of both biofilm and biofilm + GF group (last column and last row, respectively), and into Table 2 we report percentages and numbers for every pair category described with (biofilm category, biofilm + GF category).

In addition, we define the variable Global OD, for the biofilm and biofilm +GF groups, described by all OD scores of the 49 isolates considered for both. In Table 3 we report the characteristics of Global OD and Global category variables.

Finally, in Table 4 we report all statistical tests performed.

In Table 4, we performed a test of normality for the Global OD variable defined for both the biofilm and biofilm + GF groups. The test results indicate that the normality hypothesis for the Global OD variable was rejected in both cases. Consequently, the Wilcoxon test was used to compare paired data. Particularly, the Global OD score of the biofilm group displayed a mean significantly greater than that of the biofilm + GF group (*p* < 0.0001). In addition, we considered the Global category variable, because while when considering only the mean score, a reduction of score for an isolate, signals an effect of GF presence, it was not sufficient to individualize a significant change of category (Absent, Weak, Moderate and Strong). Therefore, an additional test on the Global category variable was performed. The statistical test results display a significant reduction of category from biofilm to biofilm + GF group (4 > 2, *p* < 0.0001). 

Finally, there was on analogous isolates a significant presence of isolates with category Absent in the biofilm + GF group in comparison to the biofilm group (36.7% vs. 2.0%, *p* < 0.0001), vice versa for category Strong (30.6% < 51.0%, *p* = 0.002), while for categories Weak and Moderate there were not significant differences (30.6% vs. 16.3%; 16.3% vs. 16.3%, respectively).

## 4. Discussions

Extract of mushroom or their purified antimicrobial constituents are alternative biocides that have recently gained attention as possible cleansing agents, because the chances of resistance development in bacterial cells could be minimal. *G. frondosa* has already been recognized as a mushroom with antibacterial activity, specifically the albino form as a source of natural compounds with antimicrobial properties [11,12,13].

The albino maitake mushroom is quite rare. It is distributed in temperate regions including in recent times Italy; in 2016 it was also collected for the first time in Sicily [12]. The basidioma of albino *G. frondosa* differs morphologically from the common brown/gray form, with the most significant differences being the color and shape of the pileus, the type of pores, the shape of the stipe, and the period of fructification [11,12,13].

Our preliminary laboratory experiments suggest that the cold-water extract was effective in inhibiting the growth of *S. epidermidis* and in decreasing the biofilm produced by *S. aureus* ATCC43300, a Gram-positive bacterium which could be particularly dangerous for human health, indicating possible positive use in this field. 

To our knowledge, the present study is the first report that tested an extract of albino *G. frondosa* against methicillin-resistant *S. aureus* (MRSA) biofilms that were obtained from clinical samples.

By analysis of two analogous isolate strains, we observed not only a significant reduction of OD score between the biofilm and biofilm + GF groups, but also a reduction of category. Furthermore, we observed a significant presence of isolates with strong category for the biofilm group and a significant presence of isolates with absent category for the biofilm + GF group. These results confirm that the presence of GF reduces in a significant way the presence of biofilm OD. 

In addition, our results confirm that there was a high correlation in terms of biofilm inactivation by extract of albino *G. frondosa*, as established by the crystal violet-stained optical density (at a 570-nm wavelength) readings. 

On the basis of this evidence, we believe that the extracts might be a potential lead compound in the discovery of new anti-Gram-positive and anti-biofilm agents [20,21].

The anti-biofilm property of albino *G. frondosa* extract influences specifically the bacterial adhesion and the action of antimicrobial drugs. 

In fact, having observed the role of this extract on the microbial biofilm, we suggest the use of the extract to reduce the microbial pathogenicity and to increase the activity of antimicrobial drugs [22]. Overall, the data presented in this work show a useful and up-to-date reference for further research on the constituents, properties, and functions of albino *G. frondosa* and for development and commercial applications in the form of new functional foods and therapeutic products [23].

## 5. Conclusions

Edible and medicinal fungi or mushrooms are among the most common sources of health foods and nutraceutical products. *G. frondosa* is one of the most widely explored fungal species for nutraceutical and therapeutic compounds. For a wider and more reliable application of the various components in nutraceutical and therapeutic products, it is fundamental to gain a better understanding of the structure–bioactivity relationship and the underlying mechanisms of action in the human body. Our results indicate that anti-biofilm activity of the extract of albino *G. frondosa* should be considered an important approach in assessing compounds as candidates for further development stages in antimicrobial research. In conclusion, albino *G. frondosa,* as the other form, can also be considered for its antimicrobial activities. Therefore, further experiments are needed to evaluate which are the specific components of the extract from albino *G. frondosa* involved in reducing biofilm, to improve its effect. 

## Figures and Tables

**Table 1:** Type of sample.

Type of Sample	n° of Strains
Blood	10
Catheter	12
Sputum	7
Wound	9
Urine	5
Biopsy	6
Total	49

**Table 2:** Percentages and numbers of Absent, Weak, Moderate, and Strong classifications of clinical isolates of MRSA in the biofilm and biofilm with GF groups.

	(Biofilm + GF) Group				
Biofilm Group	Absent	Weak	Moderate	Strong	Total
Absent	1 (2.0%)	0 (0.0%)	0 (0.0%)	0 (0.0%)	1 (2.0%)
Weak	13 (26.5%)	2 (4.1%)	0 (0.0%)	0 (0.0%)	15 (30.6%)
Moderate	4 (8.2%)	4 (8.2%)	0 (0.0%)	0 (0.0%)	8 (16.3%)
Strong	0 (0.0%)	2 (4.1%)	8 (16.3%)	15 (30.6%)	25 (51.0%)
Total	18 (36.7%)	8 (16.3%)	8 (16.3%)	15 (30.6%)	49

**Table 3:** Characteristics for biofilm and biofilm + GF groups of Global category and Global OD variables.

Type	Biofilm Group	(Biofilm + GF) Group
Global OD (Mean ± SD)		
Mean ± SD	0.42 ± 0.32	0.21 ± 0.17
(min-Max)	(0.04–1.13)	(0.03–0.59)
Median (CI at 95%)	0.32 (0.16–0.53)	0.12 (0.08–0.27)
Global category		
Mean ± SD	3.16 ± 0.94	2.41 ± 1.27
(min-Max)	(1–4)	(1–4)
Median (CI at 95%)	4 (3–4)	2 (1.17–3)

CI = Median Confidence interval at 95%; SD = standard deviation; min = minimum value; Max = maximum value.

**Table 4:** Statistical tests to compare the biofilm and biofilm + GF groups and their normality test.

Group	Test of Normality
Biofilm group	Global OD: *p* < 0.0001 *, (C)
Biofilm + GF group	Global OD: *p* < 0.0001 *, (C)
Type (mean and median)	biofilm vs. (biofilm +GF)
Global OD (mean)	0.42 > 0.21, *p* < 0.0001 * (W)
Global category (OD category: median)	4 > 2, *p* < 0.0001 * (W)
Type (percentages)	biofilm vs. (biofilm +GF)
Absent	2.0% < 36.7%, *p* < 0.0001 * (M)
Weak	30.6% > 16.3%, *p* = 0.18 (M)
Moderate	16.3% = 16.3%, *p* < 0.0001 * (M)
Strong	51.0% > 30.6%, *p* = 0.002 * (M)

* = significant test; C = chi square test for normality (*p* < 0.05 we assumed: reject normality); W = Wilcoxon test; M = McNemar exact test.

## Data Availability

The study did not report any data.

## Supplementary Materials

The following are available online at https://www.mdpi.com/article/10.3390/jof7070551/s1.
